# Prospective Evaluation of Positivity Rates of *Aspergillus*-Specific IgG and Quality of Life in HIV-Negative Tuberculosis Patients in Lagos, Nigeria

**DOI:** 10.3389/fcimb.2022.790134

**Published:** 2022-02-03

**Authors:** Rita O. Oladele, Titi Gbajabimiala, Nicholas Irurhe, Suzanne M. Skevington, David W. Denning

**Affiliations:** ^1^ Department of Medical Microbiology & Parasitology, College of Medicine, University of Lagos, Lagos, Nigeria; ^2^ Clinical Sciences Department, National Institute for Medical Research, Lagos, Nigeria; ^3^ Department of Radiology, College of Medicine, University of Lagos, Lagos, Nigeria; ^4^ Manchester Centre for Health Psychology, Division of Psychological Science and Mental Health, Faculty of Biology, Medicine and Health, The University of Manchester, Manchester, United Kingdom; ^5^ Manchester Fungal Infection Group, Core Technology Facility, The University of Manchester, Manchester, United Kingdom

**Keywords:** *Aspergillus* IgG, tuberculosis, quality of life, LMICs, Nigeria, chronic pulmonary aspergillosis

## Abstract

**Background:**

Pulmonary tuberculosis (PTB) often results in residual anatomical and functional changes despite microbiological cure and may be complicated by chronic pulmonary aspergillosis (CPA). In this study, we determined the perceived health-related quality of life (HRQoL) of patients during and after PTB therapy and compared it with their quantitative *Aspergillus*-specific IgG positivity rates.

**Methodology:**

We conducted a longitudinal study among TB patients attending two directly observed therapy short-course (DOTS) clinics in Lagos, Nigeria. Two hundred and four confirmed TB patients were recruited over 9 months, with five visits at baseline and 3, 6, 9, and 12 months. They were all acid-fast bacilli smear, GeneXpert, or culture positive for *Mycobacterium tuberculosis*. Two HRQoL questionnaires translated into Yoruba were self-administered. Chest X-ray and *Aspergillus* IgG were collected at each visit.

**Results:**

A total of 204 participants were recruited into this study. Most (70.6%) were age 18–39 years, and only 3.9% were above 60 years; 66.7% of all participants were males. A total of 189 (92.6%) participated in the 3-month assessment, 174 (85.3%) at 6 months, 139 (68.1%) at 9 months, and 99 (48.5%) at 12 months. At baseline, only 60.9% scored “good” or “very good” QoL and health on the WHOQOL-Bref, which improved to 77% at 6 months. At baseline, 10.4% had positive *Aspergillus* IgG levels, 15.1% at 3 months, 11.5% at 6 months, 16.7% at 9 months, and 19.3% at 12 months. Those with a positive *Aspergillus* IgG at 6 months had worse physical health (*p* = 0.001), psychological state (*p* = 0.002), social relationships (*p* = 0.006), and environmental QoL (*p* = 0.001) domains of the WHOQOL-Bref. Probable CPA was 10.4% at baseline and 19.3% at 6 months post-PTB therapy. Thirty-eight (18.6%) relocated after 6 months of treatment, 16 (7.8%) were lost to follow-up, and 11 (5.4%) died.

**Conclusion:**

Our findings reveal a significant relationship between the QoL and *Aspergillus* IgG levels of TB patients. Further follow-up studies and additional imaging are required to determine when patients develop CPA and its clinical impact.

## Introduction

Globally, tuberculosis (TB) is a major public health problem and remains one of the world’s deadliest communicable diseases. The World Health Organization (WHO) launched the “End TB Strategy” aimed at reducing TB incidence by 90% and mortality by 95% by 2030 (Uplekar et al., 2015). Each year, nearly 6 million people are diagnosed with pulmonary tuberculosis (PTB), of whom about 55% have their diagnosis confirmed with laboratory testing (World Health Organization, 2020). The recent WHO global TB report 2020 ranked Nigeria as the first in Africa and sixth globally among the 30 high TB burden countries, with a TB incidence of 219/100,000 population and 120,266 cases notified. Nigeria is on the list of 14 countries with the triple burden of TB, HIV-associated TB, and multidrug-resistant (MDR) TB with a population incidence of MDR-TB of 11/100,000 (World Health Organization, 2020).

Tuberculosis patients face various challenges such as physical restrictions, psychological and emotional issues, and economic and social problems. These known challenges impact on the quality of life of the patients (Hansel et al., 2004; Guo et al., 2009). Clinically, the therapeutic modalities, the side effects of the drugs, and the sequelae of the disease also impact on the quality of life (QoL) of the patients (Hansel et al., 2004; Muniyandi et al., 2007). PTB also results in residual anatomical and functional changes, despite microbiological cure (Pasipanodya et al., 2007). These changes are associated with post-TB lung disease (Pasipanodya et al., 2007). PTB patients often have pulmonary cavities, which can become colonized and infected by inhaled *Aspergillus* spores resulting in chronic pulmonary aspergillosis (CPA) (Denning et al., 2018). Other structural sequelae include fibrosis, chronic obstructive pulmonary disease (COPD) (Denning et al., 2018), and bronchiectasis, as well as sensitization to *Aspergillus* (IgE response), itself linked to worse lung function, independently of CPA (Dhooria et al., 2014). Worsening clinical status can be assumed to be MDR-TB.

TB services and clinical research in Nigeria have focused on the outcomes of mortality and microbiologic cure and have neglected the preferences of the patient, such as the quality of life of the patient, a crucial component of outcome. Health-related quality of life (HRQoL) involves assessing the perceptions of a person of his or her physical and mental health. Both physical and mental distress are common in TB patients leading to decreased compliance with medical care (Liefooghe et al., 1995; Rajeswari et al., 1999). The impact of PTB on HRQoL has been reported in a systematic literature review (Kastien-Hilka et al., 2016). This systematic review found that PTB had a negative impact on the HRQoL and overall wellbeing of the patients, even after PTB cure.

Quality of life assessment tools can be used in both high resource settings and in LMICs. Despite the availability of these standard instruments for assessing health-related quality of life, the feasibility, reliability, and validity of such instruments among TB patients in different populations of sub-Saharan Africa, where the burden of TB is of concern, are still limited.

This longitudinal study aimed to evaluate patient-reported HRQoL in pulmonary TB in Lagos, Nigeria, using two QoL assessment tools, namely, the generic WHOQOL-Bref (The WHOQOL Group, 1998; Skevington et al., 2004) and the specific St. George’s Respiratory Questionnaire (SGRQ) (American Thoracic Society (ATS); Welling et al., 2015). The study sought to identify persons with post-TB lung disease following “microbiological cure” 6 months post-therapy and understand the overall impact of TB on the QoL and important domains of qualities of life identified by the WHO.

## Materials and Methods

### Study Design

We conducted a longitudinal study among TB patients attending two directly observed treatment short-course (DOTS) clinics (National Institute of Medical Research and Lagos University Teaching Hospital) in Lagos, Nigeria, from March 2016 to February 2018. Ethical approval for this research was obtained from the HREC of both institutions. Confirmed (smear and/or Xpert (PCR) and/or culture positive for *Mycobacterium tuberculosis*) TB patients were recruited. Patients were excluded if they were diagnosed with multidrug-resistant TB (MDR-TB) and extensively drug-resistant TB (XDR-TB) and/or had HIV co-infection. Informed written consent was obtained from each study participant. Eligible participants received a 6-month standard TB treatment with rifampicin, isoniazid, ethambutol, and pyrazinamide. Excluded from the study were patients with extrapulmonary TB and patients with any significant associated pulmonary diseases such as lung cancer, chronic obstructive pulmonary disease, and asthma and other chronic diseases likely to affect quality of life including diabetes mellitus and cardiovascular diseases.

Two QoL assessment tools were collected: the WHOQOL-Bref and SGRQ questionnaires. Sociodemographic factors including age, gender, educational status, and work status were captured. Mean transformed QoL scores were calculated from the 26 items of the WHOQOL-Bref, for its four QoL domains (physical, psychological, social, and environment) and for general overall QoL and health (Skevington and Epton, 2018). High scores mean very good QoL. Domain scores are sensitive to change during interventions (Skevington and Epton, 2018). SGRQ is a disease-specific instrument designed to assess patients with respiratory tract and immune system diseases, especially asthma, pulmonary diseases, and COPD (American Thoracic Society (ATS); Welling et al., 2015). SGRQ comprises 50 items in three domains (symptoms, activity, and impacts on daily life). Both scores are scaled from 0 to 100, with higher scores indicating worse QoL. A minimally important difference (MID) for SGRQ was defined as an improvement of 4 points in the domain scores and the total score (American Thoracic Society (ATS); Welling et al., 2015).

Questionnaires were administered five times altogether, at the start of TB treatment and then at 3-month intervals until 6 months post-therapy (**Supplementary Table S1**). The questionnaires were incorporated into clinical care visits for the convenience of the participants and were self-administered although supervised. Patients completed the questionnaire themselves in a quiet area, free from distraction, but a research nurse was available to give explanations, if required. The questionnaire was designed to elicit the perceptions of the patient of his/her health, not others views, so family, friends, or healthcare staff did not influence the responses of the patient. An accompanying spouse or partner was asked to wait in a separate area. Similarly, patients were not allowed to take the questionnaires home. It was emphasized that they should complete the questionnaire as honestly as possible and that there are no right or wrong answers: simply the answer that best applies to them. They were asked to answer all questions. Since literacy rates in Nigeria are relatively low, translation of the questionnaires into the local dialect Yoruba was available and a nurse was available to read it aloud for those who could not read Yoruba either.

Five milliliters of blood was collected at each visit and serum was separated for *Aspergillus* IgG levels testing using ELISA (Bordier affinity products SA, Switzerland, Bordier^®^ Kit), with a cutoff of 1.0. Sputum was not collected for culture. Chest X-ray (CXR) was done at recruitment, then again at 3, 6, 9, and 12 months. Two independent radiologists, who had no immediate access to the *Aspergillus* IgG results, interpreted the CXR. CT scans were not performed and so CPA was not definitively diagnosed or confirmed. As it was highly likely in many patients, we refer to it as “probable CPA”.

### Data Analysis

The first analysis assessed measurement (psychometric properties) in the sample to find out whether the WHOQOL-Bref is a valid, reliable measure to use in this population. The second step compared the QoL of people at different intervals after starting treatment for TB. The third step compared those with and without positive *Aspergillus* antibodies, especially those with documented chronic pulmonary aspergillosis. Descriptive statistics were applied to sociodemographic data and furthermore to all HRQoL data at baseline and in all follow-up visits, to understand the HRQoL impairment of TB patients. Descriptive statistics tested the following: frequencies (*N*, *N* missing, %), central tendency (mean, median), and confidence interval (set at 95%). The distribution of data was examined by standard deviation (SD), minimum and maximum values, and frequency plots.

Significance testing (paired-samples *t*-test), multivariable analysis, and repeated measures analysis of variance (ANOVA) were based on observations that contained all data points required for a specific analysis. Overall changes in HRQoL between baseline and 6-month treatment (visit 4) were calculated as frequencies (%). Longitudinal changes were determined by the change in mean scores between all follow-up visits and baseline. The change in mean scores was examined by paired-samples *t*-test, with a statistical significance (two-tailed) set *a priori* at *p <*0.05. Changes in mean scores in the intensive treatment phase (baseline to visit 2) were compared with changes in the continuous treatment phase (visit 2 to visit 5) based on paired-samples *t*-test with a statistical significance (two-tailed) set *a priori* at *p <*0.05. The change in mean scores at each time point from baseline was also compared with the reported MID for each measure, to understand if longitudinal changes in HRQoL were clinically meaningful. A paired-samples *t*-test was applied to examine the difference between changes in mean scores and MID at a significance level *p <*0.05.

Differences in QoL mean scores among all these measures over time (baseline and follow-up visits 1–4) were examined by repeated measures ANOVA with a Bonferroni correction applied to adjust for multiple tests. Responsiveness over time for each HRQoL dimension was measured as an effect size partial eta-squared, providing information of the effect of time on changes in QoL. The resulting candidate factors were further assessed in multivariable models to understand the impact of sociodemographic factors over time [change from baseline to 6 months post-treatment (visit 5)]. The univariate and multivariate analyses included a general linear model and ANOVA.

## Results

### Sociodemographic Data

A total of 204 participants with confirmed PTB were recruited into this study, of which 144 (70.6%) were aged 18–39 years (**Table 1**). One hundred and thirty-six (66.7%) were men giving a male:female ratio of 2:1. Educational levels varied substantially (from illiteracy to tertiary education levels), with 3.9% having no formal education. About half (49.8%) of the participants were single, and 44.8% were married (**Table 1**).

**Table 1:** Sociodemographic data of the study participants.

Variable	Frequency	Percentage (%)
Age	*N* = 204	
18–39	144	70.6
40–49	36	17.6
50–59	16	7.8
>60	8	3.9
Gender	*N* = 204	
Male	136	66.7
Female	68	33.3
Educational level	*N* = 203	
None	8	3.9
Primary	33	16.3
Secondary	90	44.3
Tertiary	72	35.5
Marital status	*N* = 203	
Single	101	49.8
Married	91	44.8
Divorced	1	0.5
Widowed	2	1.0
Separated	4	2.0
Living as married	4	2.0

### Overall Quality of Life and General Health (WHOQOL-Bref)

Of the 204 participants, 189 (92.6%) participated in the baseline assessment, 174 (85.3%) at 3 months, 139 (68.1%) at 6 months, 99 (48.5%) at 9 months, and 88 (43.1%) at 12 months. Of the participants, 60.9% had a good or very good perception of their QoL at baseline assessment, 78.7% at 3 months, 77% at 6 months, 79.8% at 9 months, and 87.5% at the 12-month assessment (**Table 2**). In contrast, at baseline, 15.3% described their QoL as very poor or poor, 4.0% at 3 months, 4.4% at 6 months, 1.0 at 9 months, and 1.1% at 12 months (**Table 2**). These response trends were mirrored by general health and overall QoL questions (**Table 2**).

**Table 2:** Overall quality of life and general health.

	Very poor (%)	Poor (%)	Neither poor nor good (%)	Good (%)	Very good (%)
How would you rate your quality of life?					
Baseline	6.3	9.0	23.8	42.9	18.0
3 months	0.0	4.0	17.2	60.3	18.4
6 months	2.2	2.2	18.7	48.2	28.8
9 months	0.0	1.0	19.2	50.5	29.3
12 months	1.1	0.0	11.4	55.7	31.8
How satisfied are you with your general health?					
Baseline	12.2	25.4	27.5	26.5	8.5
3 months	1.7	13.3	19.1	50.9	15.0
6 months	2.2	9.4	19.4	51.1	18.0
9 months	3.0	3.0	22.2	46.5	25.3
12 months	4.5	3.4	11.4	50.0	30.7

### Changes in the Dimensions of HRQOL Measures

The mean scores increased with the length of TB therapy in the domains of the WHOQOL-Bref, with the highest at the 12-month assessment and the lowest during the baseline assessment (**Table 3**). Over the course of 12 months, there was significant improvement in quality of life as measured by both the WHOQOL-Bref and SGRQ. Scores decreased by 9–25 points in the SGRQ, while scores increased by 12–20 points depending on the domain of the WHOQOL-Bref.

**Table 3:** Mean scores for different domains of the HRQoL.

	Responders (*N*)	WHOQOL-Bref (mean ± SD)	SGRQ (mean ± SD)
Physical health			
Baseline	189	51.43 ± 18.90	37.89 ± 26.05
3 months	174	67.06 ± 16.40	25.86 ± 23.49
6 months	139	69.95 ± 18.81	18.33 ± 20.57
9 months	99	73.84 ± 18.18	16.93 ± 20.00
12 months	88	73.63 ± 18.23	16.83 ± 23.12
Psychological health			
Baseline	189	59.79 ± 18.29	37.89 ± 26.05
3 months	173	68.00 ± 14.58	26.03 ± 23.47
6 months	139	71.85 ± 15.00	18.33 ± 20.57
9 months	99	75.04 ± 14.11	16.93 ± 20.00
12 months	88	72.65 ± 15.76	16.83 ± 23.12
Social relationships			
Baseline	189	58.32 ± 22.24	37.89 ± 26.05
3 months	173	68.76 ± 19.98	25.98 ± 23.53
6 months	139	71.35 ± 20.91	18.33 ± 20.57
9 months	98	73.60 ± 19.89	16.93 ± 20.00
12 months	87	73.77 ± 20.74	17.18 ± 23.23
Environment			
Baseline	189	57.57 ± 16.84	37.89 ± 26.05
3 months	174	64.90 ± 14.66	25.86 ± 23.49
6 months	139	68.14 ± 15.35	18.33 ± 20.57
9 months	99	70.98 ± 14.03	16.93 ± 20.00
12 months	87	71.75 ± 14.73	15.72 ± 22.05

### Comparison of HRQoL Scores in Those With and Without a Positive *Aspergillus* IgG

**Figure 1:**
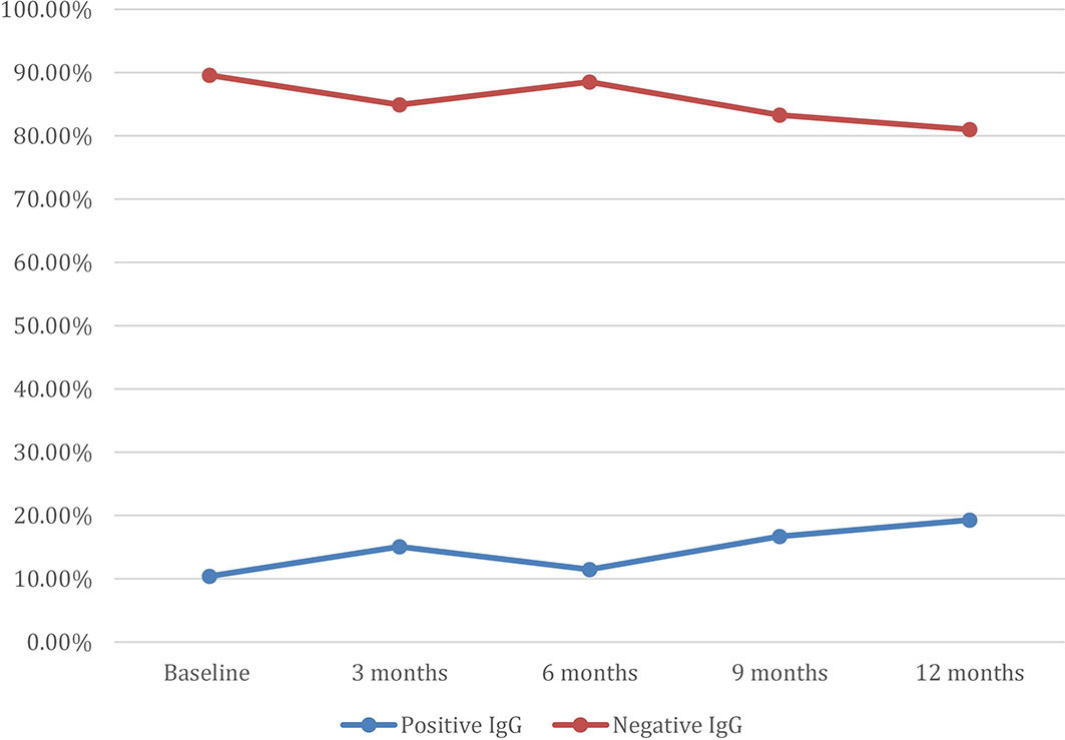
Trends in Proportion of Aspergillus IgG being positive at different time points.

Among the participants, 10.4% had a positive *Aspergillus* IgG at baseline assessment, 15.1% at 3 months, 11.5% at 6 months, 16.7% at 9 months, and 19.3% at 12 months (**Figure 1**). There was a statistically significant difference in the mean WHOQOL-Bref domain scores between participants with positive and negative *Aspergillus* IgG at the 6-month assessment, in physical health (*p* = 0.001), psychological state (*p* = 0.002), social relationships (*p* = 0.006), and environmental (*p* = 0.001) quality of life domains (**Table 4**). At 6 months, WHOQOL-Bref scores were significantly higher in patients with positive *Aspergillus* IgG levels compared with those with negative *Aspergillus* IgG levels, in all four domains (*p* < 0.05). At 12 months, QoL scores were higher in those with elevated *Aspergillus* IgG levels, compared with low *Aspergillus* IgG levels in physical, psychological, and environmental QoL domains. Although social QoL scores were lower in probable CPA patients compared with those in non-CPA patients, the difference was not significant (*p* > 0.05). To remove the confounding effect, linear regression was done using Asp IgG levels and total QoL scores, and it was statistically significant (*p*-value 0.029) at 9 months.

**Table 4:** Mean HRQoL scores in Asp IgG +ve and Asp IgG +ve participants.

Timing	Asp IgG positive	Asp IgG negative	*t*-test	*p*-value
6 months				
Physical health	81.36 ± 13.21	69.46 ± 17.86	2.754	0.016^*^
Psychological health	86.00 ± 11.88	70.81 ± 14.12	3.974	0.002^*^
Social relationships	86.36 ± 14.83	70.62 ± 20.78	3.227	0.006^*^
Environment	82.00 ± 10.31	67.04 ± 14.72	4.399	0.001^*^
12 months				
Physical health	74.09 ± 22.45	72.83 ± 18.11	0.175	0.864
Psychological health	73.27 ± 24.79	72.25 ± 14.96	0.133	0.897
Social relationships	70.00 ± 27.22	75.79 ± 17.61	−0.648	0.531
Environment	77.00 ± 15.45	72.13 ± 14.48	0.923	0.374

* Statistically significant, p < 0.05.

### Symptoms Measured by the SGRQ

Statistical difference was observed between mean symptoms scores from successive assessments over 12 months, using one-way ANOVA (*F* = 15.058, *p*-value < 0.001) (**Table 4**). A Tukey *post-hoc* test revealed that the mean baseline score was significantly higher than all the other months of assessment and significantly higher at 3 months than at 12 months (*p* < 0.05) (**Table 5**). A significant difference was also found between mean symptoms, impacts, activity, and overall score and between months of assessment (one-way ANOVA, *p* < 0.001) (**Table 5**).

**Table 5:** Mean scores of participants answering the SGRQ.

	No. of patients	Mean ± SD	*F*	*p*-value
Symptoms scores				
Baseline	196	41.55 ± 27.59	15.058	<0.001^*^
3 months	155	30.50 ± 25.29		
6 months	102	25.47 ± 23.31		
9 months	72	22.75 ± 26.89		
12 months	50	16.72 ± 22.98		
Impact scores				
Baseline	196	32.28 ± 27.51	15.121	<0.001^*^
3 months	155	21.11 ± 23.51		
6 months	102	13.72 ± 19.46		
9 months	72	13.82 ± 20.58		
12 months	50	15.49 ± 23.15		
Activity scores				
Baseline	196	39.00 ± 31.76	11.080	<0.001^*^
3 months	155	30.58 ± 29.51		
6 months	102	20.86 ± 25.88		
9 months	72	21.10 ± 25.32		
12 months	50	17.83 ± 27.41		
Overall scores				
Baseline	196	36.04 ± 26.29	16.496	<0.001^*^
3 months	155	25.53 ± 23.53		
6 months	102	17.76 ± 20.15		
9 months	72	17.48 ± 20.24		
12 months	50	16.43 ± 22.75		

***means the p-values are statistically significant.

### Comparison of SGRQ in *Aspergillus* IgG-Positive Versus *Aspergillus* IgG-Negative Participants

At 6 months, participants with positive *Aspergillus*-specific IgG levels had lower SGRQ scores indicative of worse health compared with those that had negative *Aspergillus*-specific IgG levels, but this difference was not statistically significant (*p* > 0.05) (**Table 6**). Scores were slightly higher in those with positive *Aspergillus* IgG at 12 months, but this was also not significant (*p* > 0.05).

**Table 6:** SGRQ in those with *Aspergillus* IgG-positive versus *Aspergillus* IgG-negative participants.

	IgG positive	IgG negative	*t*-test	*p*-value
6 months				
Symptoms score	23.92 ± 26.78	26.39 ± 23.93	−0.252	0.808
Impacts score	6.75 ± 10.78	15.47 ± 20.59	−1.975	0.071
Activity score	10.95 ± 21.65	23.25 ± 26.70	−1.502	0.167
Total score	10.71 ± 14.97	19.59 ± 21.08	−1.541	0.155
12 months				
Symptoms score	20.38 ± 21.30	13.36 ± 19.89	0.935	0.366
Impacts score	19.55 ± 24.16	11.03 ± 18.64	1.032	0.322
Activity score	24.14 ± 30.83	14.47 ± 24.03	0.918	0.377
Total score	21.10 ± 22.75	12.48 ± 19.05	1.097	0.293

### Radiological Findings

**Figure 2:**
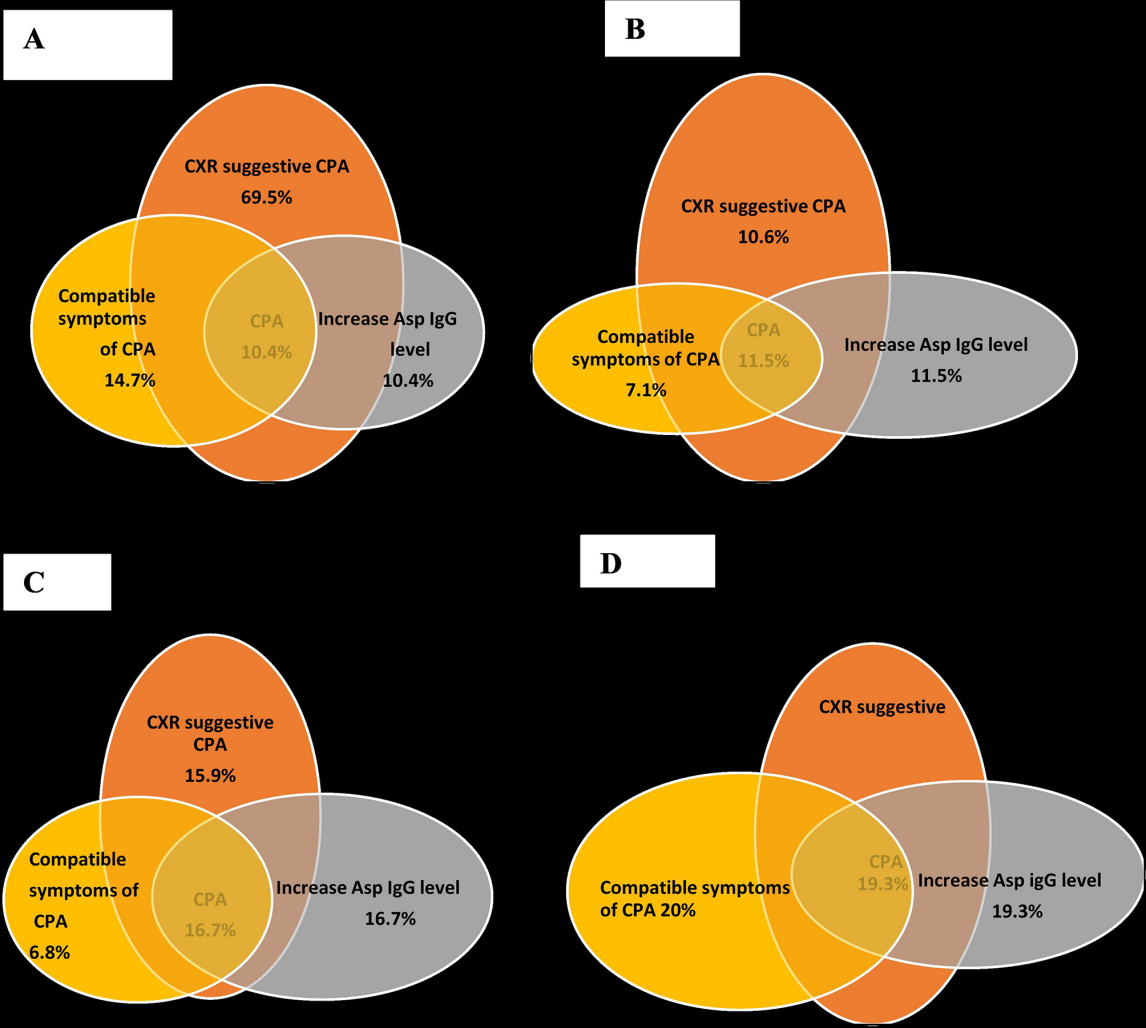
Assessment of CXR findings in studied population. **(A)** Baseline, **(B)** 6 months, **(C)** 9 months, **(D)** 12 months.

At baseline, 69.5% of the participants had CXR features reported as PTB, while 14.7% had features suggestive of CPA or PTB/CPA co-infection, and 12.4% had normal CXR (**Figure 2A**). There was improvement at 3 months on anti-TB therapy, with 41.9% showing improvement in CXR findings with only 23.4% with classical features of PTB. At 6 months when anti-TB therapy was completed, both normal CXR features and features of improvement were found in 41.2% of the participants (**Figure 2B**). Further improvement was documented at 3 months after anti-TB therapy, with normal CXR features documented in 50% (**Figure 2C**). However, at 6 months after anti-TB therapy, normal CXR features were still found in only 50% of participants, but 20% had features suggestive of either PTB or CPA or both (**Figure 2D**).

### Probable CPA

Integrating the combination of persistent symptoms (>3 months), CXR features of CPA and raised *Aspergillus* IgG enabled a diagnosis of probable CPA at different time points (**Figure 2**). Overall probable CPA was found in 20/193 (10.4%) at baseline, 17/148 (11.5%) at 6 months, and 17/88 (19.3%) at 6 months post-TB therapy. There was no statistically significant relationship between any of the domains of HRQoL and probable CPA.

## Discussion

The WHO ascertains that patient involvement in their healthcare is a social, economic, and technical necessity (Guo et al., 2009; Kastien-Hilka et al., 2016). Disease-specific health status questionnaires have proven their ability to discriminate between different levels of disease severity (Hays et al., 1995; Kastien-Hilka et al., 2016). However, generic measures like the WHOQOL-Bref (Skevington et al., 2004) are designed to be completed by patients with almost any disease or condition and may be designed to be completed by healthy people, too. Such generic instruments provide a systematic way to usefully compare patient and proxy assessments of QoL and health status across different organ systems and geographies. They also provide information beyond those obtained from clinical and microbiological parameters. Patient experience is assessed using a patient-reported outcome measure (PRO). PROs for CPA have included SGRQ, but this can only be compared among other lung diseases, not other conditions, and is poorly validated in countries with English as a second or third language.

In this study, we observed that most participants were ill at enrollment, with the lowest WHOQO-Bref and the highest SGRQ scores. Scores improved in relation to the length of TB therapy in the different QoL domains, with the highest at 12 months. Moreover, most respondents at 3 months had good perceptions of their overall QoL and general health. However, during this same period, a significant number of respondents were found to have positive *Aspergillus* IgG (**Figure 1**). Overall QoL and general health demonstrated the highest elevation of scores from baseline to 6 months. This most likely reflects the known efficacy of anti-TB therapy and the compliance of the patients.

Research studies using the HRQoL among patients treated for TB in Africa are few (Adeyeye et al., 2014), and to our knowledge, none has been longitudinal. Furthermore, this study is the first to investigate both TB and *Aspergillus* IgG levels and CPA and its effect on HRQoL. Studies like these are urgently needed (Brown et al., 2015) to better manage these large populations.

The finding of progressive improvement in QoL due to anti-TB therapy accords with the findings from similar studies that used the WHOQOL-Bref, for <12 months (Dhuria et al., 2009; Balgude and Sontakke, 2012; Aggarwal et al., 2013; Aggarwal, 2019). This was also the trend in other studies that did not use the WHOQOL-Bref (Babikako et al., 2010; Kruijshaar et al., 2010; Atif et al., 2014). All these studies revealed a gradual increase in scores and improved QoL, as treatment progressed.

Some societal factors that could influence QoL were assessed in our study. Domain scores were generally better among men, urban residents, younger patients, and those with higher socioeconomic status, less severe disease, higher education, better social security, or close family support (Duyan et al., 2005; Babikako et al., 2010; Adeyeye et al., 2014). Independent predictors of low quality of life scores included depression, illiteracy, self-stigma, low monthly income, duration of illness, concomitant illnesses, unemployment, advancing age, no family support, and sociodemographic and other economic factors (Duyan et al., 2005; Adeyeye et al., 2014). Although some of our findings are comparable to other studies, the impact of gender is not clear. We recruited more men, and being male has been associated with good domain scores (Adeyeye et al., 2014; Brown et al., 2015), although not universally (Hays et al., 1995). As expected, treatment with antituberculosis drugs improves the HRQoL of patients (Deribew et al., 2009). From a programmatic perspective, it is important to ensure adherence to medications and avoid treatment default.

In our study, the 12-month means of physical and psychological QoL in this study were lower than the means at the 9-month visit. This finding was previously reported in a study by Mamani et al. in 2014 (Mamani et al., 2014) and may be attributed to a recurrence of symptoms. If so, these symptoms could be due to the re-emergence of TB or the presence of aspergillosis; this is further supported by CXR reports (**Figures 2B, C** **)**. Alternatively, it could represent co-infection of TB and aspergillosis, bronchiectasis, or *Aspergillus* bronchitis complicating bronchiectasis. Further studies involving fungal culture and chest imaging would be helpful to measure the prevalence of pulmonary aspergillosis complicating tuberculosis among this cohort.

There was, however, a significant difference (*p* < 0.05) between respondents with positive and negative *Aspergillus* IgG at 6 months of assessment, in all the domains of the WHOQOL-Bref. This could result from the continuous presence of symptoms, as reported by a previous longitudinal study of CPA (Bongomin et al., 2018). A Ugandan study has reported that *Aspergillus*-specific IgG antibodies were elevated in 4% of HIV-infected adults at the start of TB treatment and in 9% at the end (Kwizera et al., 2017). This is likely due to colonization and either the development of CPA, a reflection of *Aspergillus* co-infection that needs to be resolved, or *Aspergillus* bronchitis complicating bronchiectasis among TB patients. Given the slow genesis of CPA, longitudinal follow-up and CT scanning are required, but our radiological data appear to supports this hypothesis (**Figure 2**). Reduced pulmonary function persists in patients cured of TB (Pasipanodya et al., 2007).

A drop in the number of respondents with each visit was observed in this study. Reasons include transfers of patients to sites closer to home, treatment default, death, and hospitalization. Other studies have reported that the dropout at the second and third follow-up visits was mostly among younger patients with no physical impairment as well as among male patients and due to poverty, severe psychological distress, or alcohol misuse (Louw et al., 2012; Atif et al., 2014; Olufemi et al., 2018). It is important to note that the treatment of TB was completed for these patients at 6 months. Thus, the need to return to the clinic may have not been vital to them visiting the facility again, as they may have achieved a complete cure to the disease. Also with improved QoL, there is a higher likelihood that treatment default may be high due to reduced motivation to improve it.

Some limitations of the study were the continuous drop in respondent numbers at 9 and 12 months, which may have had some impact on the results. A defined cutoff for *Aspergillus* IgG levels has not been established for Africans, although the European-derived cutoff (Wilopo et al., 2020) performs well in Vietnam (Nguyen et al., 2021). CT scan, which is a better representative imaging tool, was not used due to cost, and its inclusion would have allowed confirmation of the diagnosis of CPA. Also, patients underwent HIV testing when diagnosed with TB before starting treatment. HIV testing was not repeated during the treatment and any change in HIV status was therefore unknown. The severity of PTB was not accessed since it was not the focus of the study.

In conclusion, different domains of HRQoL can be a helpful tool for the assessment of the patient and outcome prediction. HRQoL is hindered in patients with PTB and improves significantly with program-based treatment. HRQoL-based disease appraisals in resource-limited countries have become an important instrument to grasp health outcomes and provide focused and empirically informed ways to manage care and treatment better. *Aspergillus* IgG levels were significantly raised in patients being managed for PTB, probably reflecting colonization by *Aspergillus* or early CPA, which alters treatment outcomes among TB patients. Routine screening of *Aspergillus* IgG at the DOTS facility would assist with improving the QoL of patients who may have been misdiagnosed as having PTB. Further follow-up studies and additional imaging, preferably CT scan, are required to determine when these patients develop CPA and its clinical impact.

## Data Availability Statement

The raw data supporting the conclusions of this article will be made available by the authors, without undue reservation.

## Ethics Statement

The studies involving human participants were reviewed and approved by the Human Research Ethics Committee (HREC), Lagos University Teaching Hospital, Lagos, Nigeria; HREC, National Institute of Medical Research, Lagos, Nigeria; and HREC, The University of Manchester. The patients/participants provided their written informed consent to participate in this study.

## Author Contributions

RO, SS, and DD conceptualized and designed the study. RO, TG, and NI collated the data. RO, SS, and DD analyzed and interpreted the data. All authors contributed to the writing and review of the manuscript.

## Funding

This work was supported with funds from University Hospital South Manchester Charity Trust (Grant award number: TREG 006).

## Conflict of Interest

The authors declare that the research was conducted in the absence of any commercial or financial relationships that could be construed as a potential conflict of interest.

## Publisher’s Note

All claims expressed in this article are solely those of the authors and do not necessarily represent those of their affiliated organizations, or those of the publisher, the editors and the reviewers. Any product that may be evaluated in this article, or claim that may be made by its manufacturer, is not guaranteed or endorsed by the publisher.

## References

[B1] Adeyeye O. O. Ogunleye O. O. Coker A. Kuyinu Y. Bamisile R. T. Ekrikpo. (2014). Factors Influencing Quality of Life and Predictors of Low Quality of Life Scores in Patients on Treatment for Pulmonary Tuberculosis: A Cross Sectional Study. J. Public Health Afr. 5 (2), 88–92. doi: 10.4081/jphia.2014.366 PMC5345416 28299129

[B2] Aggarwal A. N. (2019). Quality of Life With Tuberculosis. J. Clin. Tuberc. Other Mycobact. Dis. 17, 100121. doi: 10.1016/j.jctube.2019.100121 31788563 PMC6880022

[B3] Aggarwal A. N. Gupta D. Janmeja A. K. Jindal S. K. (2013). Assessment of Health-Related Quality of Life in Patients With Pulmonary Tuberculosis Under Programme Conditions. Int. J. Tuberc. Lung Dis. 17 (7), 947–953. doi: 10.5588/ijtld.12.0299 23743314

[B4] Atif M. Sulaiman S. A. Shafie A. A. Asif M. Sarfraz M. K. Low H. C. (2014). Impact of Tuberculosis Treatment on Health-Related Quality of Life of Pulmonary Tuberculosis Patients: A Follow-Up Study. Health Qual. Life Outcomes 12, 19. doi: 10.1186/1477-7525-12-19 24528499 PMC3925792

[B5] American Thoracic Society (ATS). St. George’s Respiratory Questionnaire (SGRQ). Available at: https://www.thoracic.org/members/assemblies/assemblies/srn/questionaires/sgrq.php.

[B6] Babikako H. M. Neuhauser D. Katamba A. Mupere E. (2010). Feasibility, Reliability and Validity of Health-Related Quality of Life Questionnaire Among Adult Pulmonary Tuberculosis Patients in Urban Uganda: Cross-Sectional Study. Health Qual. Life Outcomes 8, 93. doi: 10.1186/1477-7525-8-93 20813062 PMC2944342

[B7] Balgude A. Sontakke S. (2012). Study of Impact of Antitubercular Therapy on Quality of Life. Indian J. Med. Sci. 66 (3-4), 71–77. doi: 10.4103/0019-5359.110911 23603624

[B8] Bongomin F. Harris C. Hayes G. Kosmidis C. Denning D. W. (2018). Twelve-Month Clinical Outcomes of 206 Patients With Chronic Pulmonary Aspergillosis. PloS One 13 (4), e0193732. doi: 10.1371/journal.pone.0193732 29634721 PMC5892866

[B9] Brown J. Capocci S. Smith C. Morris S. Abubakar I. Lipman M. (2015). Health Status and Quality of Life in Tuberculosis. Int. J. Infect. Dis. 32, 68–75. doi: 10.1016/j.ijid.2014.12.045 25809759

[B10] Denning D. W. Page I. D. Chakaya J. Jabeen K. Jude C. M. Cornet M.. (2018). Case Definition of Chronic Pulmonary Aspergillosis in Resource-Constrained Settings. Emerging Infect. Dis. 24 (8). doi: 10.3201/eid2408.171312 PMC6056117 30016256

[B11] Deribew A. Tesfaye M. Hailmichael Y. Negussu N. Daba S. Wogi A. (2009). Tuberculosis and HIV Co-Infection: Its Impact on Quality of Life. Health Qual. Life Outcomes 7, 105. doi: 10.1186/1477-7525-7-105 20040090 PMC2809048

[B12] Dhooria S. Kumar P. Saikia B. Aggarwal A. Gupta D. Behera D.. (2014). Prevalence of Aspergillus Sensitisation in Pulmonary Tuberculosis-Related Fibrocavitary Disease. Int. J. Tuberc. Lung Dis. 18 (7), 850–855. doi: 10.5588/ijtld.13.0838 24902565

[B13] Dhuria M. Sharma N. Narender P. S. Ram C. J. Saha R. Gopal K. I. (2009). A Study of the Impact of Tuberculosis on the Quality of Life and the Effect After Treatment With DOTS. Asia Pac. J. Public Health 21 (3), 312–320. doi: 10.1177/1010539509336242 19443879

[B14] Duyan V. Kurt B. Aktas Z. Duyan G. C. Kulkul D. O. (2005). Relationship Between Quality of Life and Characteristics of Patients Hospitalised With Tuberculosis. Int. J. Tuberc. Lung Dis. 9 (12), 1361–1366. 16466059

[B15] Guo N. Marra F. Marra C. A. (2009). Measuring Health-Related Quality of Life in Tuberculosis: A Systematic Review. Health Qual. Life Outcomes 7, 14. doi: 10.1186/1477-7525-7-14 19224645 PMC2651863

[B16] Hansel N. N. Wu A. W. Chang B. Diette G. B. (2004). Quality of Life in Tuberculosis: Patient and Provider Perspectives. Qual. Life Res. 13, 639–652. doi: 10.1023/B:QURE.0000021317.12945.f0 15130027

[B17] Hays R. D. Wells K. B. Sherbourne C. D. Rogers W. Spritzer K. (1995). Functioning and Well-Being Outcomes of Patients With Depression Compared With Chronic General Medical Illnesses. Arch. Gen. Psychiatry 52 (1), 11–19. doi: 10.1001/archpsyc.1995.03950130011002 7811158

[B18] Kastien-Hilka T. Abulfathi A. Rosenkranz B. Bennett B. Schwenkglenks M. Sinanovic E. (2016). Health-Related Quality of Life and Its Association With Medication Adherence in Active Pulmonary Tuberculosis–a Systematic Review of Global Literature With Focus on South Africa. Health Qual. Life Outcomes 14 (1), 1–3. doi: 10.1186/s12955-016-0442-6 26969306 PMC4788905

[B19] Kruijshaar M. E. Lipman M. Essink-Bot M. L. Lozewicz S. Creer D. Dart S. (2010). Health Status of UK Patients With Active Tuberculosis. Int. J. Tuberc. Lung Dis. 14 (3), 296–302. 20132620

[B20] Kwizera R. Parkes-Ratanshi R. Page I. D. Sekaggya-Wiltshire C. Musaazi J. Fehr J.. (2017). Elevated Aspergillus-Specific Antibody Levels Among HIV Infected Ugandans With Pulmonary Tuberculosis. BMC Pulm. Med. 17 (1), 1–9. doi: 10.1186/s12890-017-0500-9 29162063 PMC5699185

[B21] Liefooghe R. Michiels N. Habib S. Moran M. B. De Muynck A. (1995). Perception and Social Consequences of Tuberculosis: A Focus Group Study of Tuberculosis Patients in Sialkot, Pakistan. Soc. Sci. Med. 41 (12), 1685–1692. doi: 10.1016/0277-9536(95)00129-U 8746868

[B22] Louw J. Peltzer K. Naidoo P. Matseke G. Mchunu G. Tutshana B. (2012). Quality of Life Among Tuberculosis (TB), TB Retreatment and/or TB-HIV Co-Infected Primary Public Health Care Patients in Three Districts in South Africa. Health Qual. Life Outcomes 10, 77. doi: 10.1186/1477-7525-10-77 22742511 PMC3422995

[B23] Mamani M. Majzoobi M. M. Ghahfarokhi S. M. Esna-Ashari F. Keramat F. (2014). Assessment of Health-Related Quality of Life Among Patients With Tuberculosis in Hamadan, Western Iran. Oman Med. J. 29 (2), 102–105. doi: 10.5001/omj.2014.25 24715935 PMC3976728

[B24] Muniyandi M. Rajeswari R. Balasubramanian R. Nirupa C. Gopi P. G. Jaggarajamma K.. (2007). Evaluation of Post-Treatment Health-Related Quality of Life (HRQoL) Among Tuberculosis Patients. Int. J. Tuberc. Lung Dis. 11, 887–892. 17705955

[B25] Nguyen N. T. Le Ngoc H. Nguyen N. V. Dinh L. V. Nguyen H. V. Nguyen H. T.. (2021). Chronic Pulmonary Aspergillosis Situation Among Post Tuberculosis Patients in Vietnam: An Observational Study. J. Fungi 7 (7), 532. doi: 10.3390/jof7070532 PMC8307285 34209322

[B26] Olufemi A. O. Chikaodinaka A. A. Abimbola P. Oluwatoyin A. T. Oluwafunmilola A. Fasanmi K. T. (2018). Health-Related Quality of Life (HRQoL) Scores Vary With Treatment and may Identify Potential Defaulters During Treatment of Tuberculosis. Malawi Med. J. 30 (4), 283–290. doi: 10.4314/mmj.v30i4.12 31798808 PMC6863409

[B27] Pasipanodya J. G. Miller T. L. Vecino M. Munguia G. Bae S. Drewyer G.. (2007). Using the St. George Respiratory Questionnaire to Ascertain Health Quality in Persons With Treated Pulmonary Tuberculosis. Chest 132, 1591–1598. doi: 10.1378/chest.07-0755 17890471

[B28] Rajeswari R. Balasubramanian R. Muniyandi M. Geetharamani S. Thresa X. Venkatesan P. (1999). Socio-Economic Impact of Tuberculosis on Patients and Family in India. Int. J. Tuberc. Lung Dis. 3 (10), 869–877. 10524583

[B29] Skevington S. M. Epton T. (2018). How Will the Sustainable Development Goals Deliver Changes in Wellbeing? A Systematic Review and Meta-Analysis to Investigate Whether WHOQOL-BREF Scores Respond to Change. Br. Med. J. Global Health 3, 1–13, 0e.000609. doi: 10.1136/bmjgh-2017-000609 PMC5759710 29379649

[B30] Skevington S. M. Lotfy M. O’Connell K. A. (2004). The World Health Organisation's WHOQOL-BREF Quality of Life Assessment: Psychometric Properties and Results of the International Field Trial- A Report From the WHOQOL Group. Qual. Life Res. 13 (2), 299–310. doi: 10.1023/B:QURE.0000018486.91360.00 15085902

[B31] The WHOQOL Group (1998). Development of the WHOQOL-BREF Quality of Life Assessment. Psychol. Med. 28, 551–558. doi: 10.1017/S0033291798006667 9626712

[B32] Uplekar M. Weil D. Lonnroth K. Jaramillo E. Lienhardt C. Dias H. M.. (2015). WHO's New End TB Strategy. Lancet 385 (9979), 1799–1801. doi: 10.1016/S0140-6736(15)60570-0 25814376

[B33] Welling J. B. Hartman J. E. Ten Hacken N. H. Klooster K. Slebos D. J. (2015). The Minimal Important Difference for the St George's Respiratory Questionnaire in Patients With Severe COPD. Eur. Respir. J. 46 (6), 1598–1604. doi: 10.1183/13993003.00535-2015 26493797

[B34] Wilopo B. A. Hunter E. S. Richardson M. D. Denning D. W. (2020). Optimising the Cut-Off of the Bordier Aspergillus IgG ELISA for the Diagnosis of Chronic Pulmonary Aspergillosis. J. Microbiol. Methods 176, 106021. doi: 10.1016/j.mimet.2020.106021 32795637

[B35] World Health Organization. (2020). Global Tuberculosis Report 2020 (Geneva: World Health Organization).

