# Supplementary material for: Prospective Evaluation of Positivity Rates of Aspergillus-Specific IgG and Quality of Life in HIV-Negative Tuberculosis Patients in Lagos, Nigeria
Source: Front Cell Infect Microbiol. 2022 Feb 3;12:790134. doi: 10.3389/fcimb.2022.790134 (PMC8851390; doi:10.3389/fcimb.2022.790134)
Supplement: Supplementary file 1 [file DataSheet_1.docx]

**Oladele et al**

**Supplementary data**

**MATERIALS and METHODS**

Table S1. The tests done and research tools administered over the course of the study.

| Test | Baseline | 2-3months | 6months, (completion of treatment) | 9months | 12months |
| --- | --- | --- | --- | --- | --- |
| WHOQoL | X | X | X | X | X |
| SGRQ | X | X | X | X | X |
| Weight (Kg) | X | X | X | X | X |
| Aspergillus IgG | X | X | X | X | X |
| Chest Xray | X | X | X |  | X |

**Table 4.5.1: PHYSICAL HEALTH**

|  | **Frequency** | **Mean±SD** | **t-test** | **p-value** |
| --- | --- | --- | --- | --- |
| **Baseline** |  |  |  |  |
| Positive IgG | 19 | 49.26±17.25 | -0.512 | 0.613 |
| Negative IgG | 154 | 51.42±18.47 |  |  |
| **3 months** |  |  |  |  |
| Positive IgG | 24 | 63.54±3.32 | -1.091 | 0.248 |
| Negative IgG | 139 | 67.48±1.40 |  |  |
| **6 months** |  |  |  |  |
| Positive IgG | 11 | 81.36±13.21 | 2.754 | 0.001* |
| Negative IgG | 113 | 69.46±17.86 |  |  |
| **9 months** |  |  |  |  |
| Positive IgG | 14 | 80.50±13.63 | 1.708 | 0.102 |
| Negative IgG | 73 | 17.43±17.43 |  |  |
| **12 months** |  |  |  |  |
| Positive IgG | 11 | 74.10±22.44 | 0.175 | 0.864 |
| Negative IgG | 53 | 72.83±18.11 |  |  |

*There is a statistically significant difference, P<0.05.

**Table 4.5.2: PSYCHOLOGICAL HEALTH**

|  | **Frequency** | **Mean±SD** | **t-test** | **p-value** |
| --- | --- | --- | --- | --- |
| **Baseline** |  |  |  |  |
| Positive IgG | 19 | 59.84±18.94 | 0.127 | 0.900 |
| Negative IgG | 154 | 59.26±18.54 |  |  |
| **3 months** |  |  |  |  |
| Positive IgG | 24 | 66.13±13.86 | -0.566 | 0.575 |
| Negative IgG | 138 | 67.88±15.03 |  |  |
| **6 months** |  |  |  |  |
| Positive IgG | 11 | 86.00±11.88 | 3.974 | 0.002* |
| Negative IgG | 113 | 70.81±14.12 |  |  |
| **9 months** |  |  |  |  |
| Positive IgG | 14 | 73.29±16.26 | -0.354 | 0.728 |
| Negative IgG | 73 | 74.93±14.14 |  |  |
| **12 months** |  |  |  |  |
| Positive IgG | 11 | 73.27±24.79 | 0.133 | 0.897 |
| Negative IgG | 53 | 72.24±14.96 |  |  |

*There is a statistically significant difference, P<0.05.

**Table 4.5.3: SOCIAL RELATIONSHIPS**

|  | **Frequency** | **Mean±SD** | **t-test** | **p-value** |
| --- | --- | --- | --- | --- |
| **Baseline** |  |  |  |  |
| Positive IgG | 19 | 60.79±20.56 | 0.572 | 0.573 |
| Negative IgG | 154 | 57.90±22.35 |  |  |
| **3 months** |  |  |  |  |
| Positive IgG | 24 | 65.92±17.17 | -0.839 | 0.407 |
| Negative IgG | 138 | 69.21±20.93 |  |  |
| **6 months** |  |  |  |  |
| Positive IgG | 11 | 86.36±14.83 | 3.227 | 0.006* |
| Negative IgG | 113 | 70.62±20.78 |  |  |
| **9 months** |  |  |  |  |
| Positive IgG | 14 | 72.71±11.70 | -0.602 | 0.551 |
| Negative IgG | 73 | 75.11±21.01 |  |  |
| **12 months** |  |  |  |  |
| Positive IgG | 10 | 77.00±27.23 | -0.648 | 0.531 |
| Negative IgG | 53 | 72.13±17.62 |  |  |

*There is a statistically significant difference, P<0.05.

**Table 4.5.4: ENVIRONMENT**

|  | **Frequency** | **Mean±SD** | **t-test** | **p-value** |
| --- | --- | --- | --- | --- |
| **Baseline** |  |  |  |  |
| Positive IgG | 19 | 58.63±16.29 | 0.342 | 0.735 |
| Negative IgG | 154 | 57.26±17.30 |  |  |
| **3 months** |  |  |  |  |
| Positive IgG | 24 | 63.17±13.24 | -0.831 | 0.412 |
| Negative IgG | 139 | 65.65±14.94 |  |  |
| **6 months** |  |  |  |  |
| Positive IgG | 11 | 82.00±10.31 | 4.399 | 0.001* |
| Negative IgG | 113 | 67.04±14.72 |  |  |
| **9 months** |  |  |  |  |
| Positive IgG | 14 | 72.00±9.35 | 0.078 | 0.939 |
| Negative IgG | 73 | 71.77±14.07 |  |  |
| **12 months** |  |  |  |  |
| Positive IgG | 10 | 77.00±15.46 | 0.923 | 0.374 |
| Negative IgG | 53 | 72.13±14.48 |  |  |

*There is a statistically significant difference, P<0.05.

Index Aspergillus.IgG values of participants

**Table 1.1: Post hoc’s analysis of the mean difference in symptoms scores at the months of assessment**

|  |  | **Mean difference** | **p-value** |
| --- | --- | --- | --- |
| Baseline | 3 months | 11.05 | 0.001* |
|  | 6 months | 16.08 | <0.001* |
|  | 9 months | 18.80 | <0.001* |
|  | 12 months | 24.83 | <0.001* |
| 3months | Baseline | -11.06 | 0.001* |
|  | 6 months | 5.02 | 0.545 |
|  | 9 months | 7.74 | 0.219 |
|  | 12 months | 13.77 | 0.010* |
| 6months | Baseline | -16.08 | <0.001* |
|  | 3 months | -5.02 | 0.545 |
|  | 9 months | 2.72 | 0.960 |
|  | 12 months | 8.75 | 0.285 |
| 9months | Baseline | -18.80 | <0.001* |
|  | 3 months | -7.74 | 0.219 |
|  | 6 months | -2.72 | 0.960 |
|  | 12 months | 6.03 | 0.709 |
| 12months | Baseline | -24.83 | 0.000* |
|  | 3 months | -13.77 | 0.010* |
|  | 6 months | -8.75 | 0.285 |
|  | 9 months | -6.03 | 0.709 |

*Difference is statistically significant, p<0.05.

**Table 2.1: Post hoc’s analysis of the mean impacts scores at the months of assessment**

|  |  | **Mean difference** | **p-value** |
| --- | --- | --- | --- |
| Baseline | 3months | 11.16 | <0.001* |
|  | 6months | 18.56 | <0.001* |
|  | 9months | 18.46 | <0.001* |
|  | 12months | 16.78 | <0.001* |
| 3months | Baseline | -11.16 | <0.001* |
|  | 6months | 7.40 | 0.111 |
|  | 9months | 7.30 | 0.206 |
|  | 12months | 5.62 | 0.600 |
| 6months | Baseline | -18.56 | <0.001* |
|  | 3months | -7.40 | 0.111 |
|  | 9months | -0.10 | 1.000 |
|  | 12months | -1.77 | 0.993 |
| 9months | Baseline | -18.46 | <0.001* |
|  | 3months | -7.30 | 0.206 |
|  | 6months | 0.10 | 1.000 |
|  | 12months | -1.68 | 0.996 |
| 12months | Baseline | -16.78 | <0.001* |
|  | 3months | -5.62 | 0.600 |
|  | 6months | 1.77 | 0.993 |
|  | 9months | 1.67 | 0.996 |

*difference s statistically significant, p-value <0.05

**Table 3.1: Post hoc’s analysis of the mean activity scores at the months of assessment**

|  |  | **Mean difference** | **p-value** |
| --- | --- | --- | --- |
| Baseline | 3months | 8.42 | 0.56 |
|  | 6months | 18.14 | <0.001* |
|  | 9months | 17.90 | <0.001* |
|  | 12months | 21.17 | <0.001* |
| 3months | Baseline | -8.42 | 0.056 |
|  | 6months | 9.72 | 0.067 |
|  | 9months | 9.49 | 0.149 |
|  | 12months | 12.76 | 0.055 |
| 6months | Baseline | -18.14 | <0.001* |
|  | 3months | -9.72 | 0.067 |
|  | 9months | -0.24 | 1.000 |
|  | 12months | 3.03 | 0.974 |
| 9months | Baseline | -17.90 | <0.001* |
|  | 3months | -9.49 | 0.149 |
|  | 6months | 0.24 | 1.000 |
|  | 12months | 3.27 | 0.973 |
| 12months | Baseline | -21.17 | <0.001* |
|  | 3months | -12.76 | 0.055 |
|  | 6months | -3.03 | 0.974 |
|  | 9months | -3.27 | 0.973 |

*difference is statistically significant, p-value<0.05

**Table 4.1: Post hoc’s analysis of the mean overall scores at the months of assessment**

|  |  | **Mean difference** | **p-value** |
| --- | --- | --- | --- |
| Baseline | 3months | 10.51 | <0.001* |
|  | 6months | 18.28 | <0.001* |
|  | 9months | 18.56 | <0.001* |
|  | 12months | 19.61 | <0.001* |
| 3months | Baseline | -10.51 | <0.001* |
|  | 6months | 7.78 | 0.073 |
|  | 9months | 8.05 | 0.117 |
|  | 12months | 9.10 | 0.123 |
| 6months | Baseline | -18.28 | <0.001* |
|  | 3months | -7.78 | 0.073 |
|  | 9months | 0.27 | 1.000 |
|  | 12months | 1.32 | 0.998 |
| 9months | Baseline | -18.56 | <0.001* |
|  | 3months | -8.05 | 0.117 |
|  | 6months | -0.27 | 1.000 |
|  | 12months | 1.05 | 0.999 |
| 12months | Baseline | -19.61 | <0.001* |
|  | 3months | -9.10 | 0.123 |
|  | 6months | -1.32 | 0.998 |
|  | 9months | -1.05 | 0.999 |

*difference is statistically significant, p-value<0.05
