# Supplementary material for: Prospective Evaluation of Positivity Rates of Aspergillus-Specific IgG and Quality of Life in HIV-Negative Tuberculosis Patients in Lagos, Nigeria
Source: Front Cell Infect Microbiol. 2022 Feb 3;12:790134. doi: 10.3389/fcimb.2022.790134 (PMC8851390; doi:10.3389/fcimb.2022.790134)
Supplement: Supplementary file 2 [file Table_1.docx]

OVERALL HRQOL BETWEEN CPA AND NON-CPA PATIENTS

|  | **CPA Mean±SD** | **NON-CPA Mean±SD** | **t-test** | **p-value** |
| --- | --- | --- | --- | --- |
| **Physical health** | 69.75±13.49 | 66.91±8.99 | 0.392 | 0.705 |
| **Psychological health** | 71.71±9.76 | 69.02±6.02 | 0.523 | 0.615 |
| **Social relationship** | 72.56±9.90 | 68.99±6.58 | 0.670 | 0.522 |
| **Environment** | 70.56±9.64 | 66.77±6.03 | 0.746 | 0.477 |
