# Supplementary material for: Prospective Evaluation of Positivity Rates of Aspergillus-Specific IgG and Quality of Life in HIV-Negative Tuberculosis Patients in Lagos, Nigeria
Source: Front Cell Infect Microbiol. 2022 Feb 3;12:790134. doi: 10.3389/fcimb.2022.790134 (PMC8851390; doi:10.3389/fcimb.2022.790134)
Supplement: Supplementary file 3 [file Table_2.docx]

**RESULTS**

**Linear regression of Total QOL scores and Aspergillus IgG index in study participants**

|  | **Constant** | **Beta** | **CI (95%)** | **P-value** |
| --- | --- | --- | --- | --- |
| **Baseline** | 36.001 | -0.010 | -0.036 – 0.015 | 0.427 |
| **3 months** | 26.589 | -0.648 | -9.054 – 7.758 | 0.879 |
| **6 months** | 20.794 | -4.800 | -13.864 – 4.265 | 0.296 |
| **9 months** | 13.622 | 9.002 | 0.945 – 17.059 | 0.029* |
| **12 months** | 15.827 | 2.148 | -6.361 – 10.658 | 0.614 |

*Statistically significant, p<0.05

Prediction equation

QOL= Constant + Beta (Aspergillus IgG index)
